# Non-absorbable mesh erosion following hiatal repair: a case series

**DOI:** 10.1093/jscr/rjag067

**Published:** 2026-02-14

**Authors:** Catherine Jenn Yi Cheang, Jessica Yan-Seen Ng, Daniel Leonard Chan, Garett Smith

**Affiliations:** Department of Upper Gastrointestinal Surgery, Royal North Shore Hospital, Reserve Road, St Leonards, New South Wales 2065, Australia; Department of Upper Gastrointestinal Surgery, Princess Alexandra Hospital, 199 Ipswich Road, Woolloongabba QLD 4102, Australia; Department of Upper Gastrointestinal Surgery, Royal North Shore Hospital, Reserve Road, St Leonards, New South Wales 2065, Australia; Department of Upper Gastrointestinal Surgery, Princess Alexandra Hospital, 199 Ipswich Road, Woolloongabba QLD 4102, Australia; St George and Sutherland Clinic School, Faculty of MedicIne & Health, University of New South Wales, Sydney, Australia; Department of Upper Gastrointestinal Surgery, Royal North Shore Hospital, Reserve Road, St Leonards, New South Wales 2065, Australia; Department of Upper Gastrointestinal Surgery, Princess Alexandra Hospital, 199 Ipswich Road, Woolloongabba QLD 4102, Australia

**Keywords:** hiatal hernia (HH), light weight polypropylene mesh (LWPM), mesh erosion, hiatal repair

## Abstract

Despite an absence of recent supportive evidence, mesh is frequently used as an adjunct to suture closure during hiatal hernia repair to increase repair durability. Erosion of non-absorbable mesh is a well-recognized complication of this approach. We present a case series of three patients with asymptomatic erosion of light weight polypropylene mesh (LWPM). From a series of 393 patients undergoing hiatal repair with LWPM, three cases of mesh erosion were identified. One male presented with ongoing reflux symptoms and recurrent hiatal failure who ultimately underwent Roux-en-Y gastric bypass with resolution of reflux symptoms. Two females presented with asymptomatic erosion. One underwent gastroscopy and colonoscopy for investigation of iron deficiency and the other gastroscopy for bleeding gastric ulceration unrelated to mesh erosion. Mesh erosion is a well-recognized and feared complication of mesh-augmented hiatal repair. Erosion of LWPM may be associated with fewer symptomatic implications than previously anticipated.

## Introduction

The use of prosthetic mesh to augment suture repair of large paraoesophageal hernias is common but remains controversial. Several meta-analyses have demonstrated that crural reinforcement with non-absorbable mesh may improve the durability of repair for large hiatus hernias [1–5]. However, recent randomized controlled trials have not shown a clear benefit in reducing recurrence when absorbable or non-absorbable mesh is used compared to suture repair for hiatus hernias [6, 7]. While mesh-specific complications are rare, they can be severe and may include cardiac injury during fixation, mesh erosion requiring surgical resection of the foregut, and oesophageal stenosis caused by fibrosis [8]. The true incidence of these complications remains unclear due to likely underreporting. Estimated rates from surveys [9] and systematic reviews [2] suggest erosion and stricture rates of ~0.2%–0.5%, but likely underestimate the real-world burden. We describe three cases of non-absorbable mesh erosion following hiatal repair.

## Case series

Between 2005 and 2017, 393 patients underwent surgical repair of large paraoesophageal hernias at our institution using a standardized operative technique. The median age of the cohort was 71 years (31–93), with a female predominance of 74%. All patients underwent the same operative technique, consisting of hernia sac dissection, posterior sutured cruroplasty, and reinforcement with an onlay TiMesh. The mesh was secured with non-absorbable helical ProTack screws. Routine postoperative endoscopic surveillance was not performed. We present three patients (0.76%) with mesh erosion, diagnosed incidentally or during investigations of unrelated upper gastrointestinal symptoms. Notably, none of these patients required mesh explantation, and all were managed conservatively.

### Case 1

A 61-year-old male with hypertension, hypercholesterolaemia, and gastro-oesophageal reflux disease had previously undergone laparoscopic fundoplication 10 years prior to presenting with recurrent reflux symptoms and a hiatal hernia (HH) recurrence. During revision surgery, TiMesh was placed to reinforce the hiatal repair. He re-presented 3.5 years later with recurrent reflux symptoms, at which time mesh erosion was identified on gastroscopy. Due to persistent reflux symptoms, he subsequently underwent a Roux-en-Y gastric bypass 13 years post-TiMesh repair. The mesh was not explanted during this procedure, and his symptoms have since improved.

### Case 2

**Figure 1:**
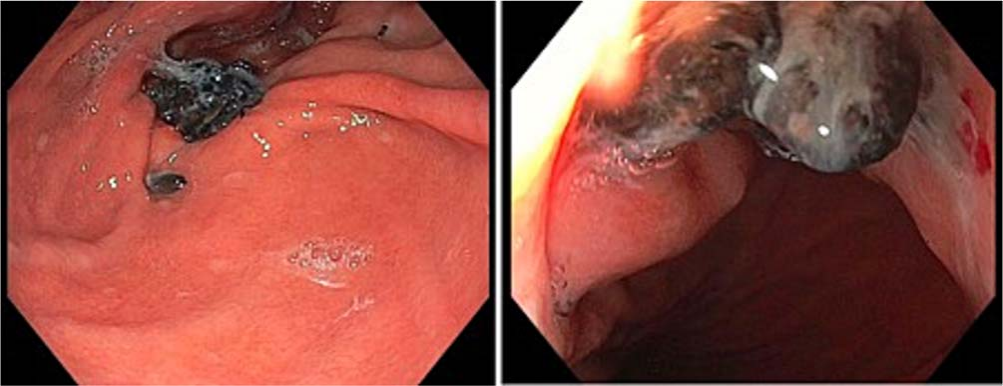
Gastroscopy images of gastric cardia with visible sutures and mesh with associated adjacent mucosal erosion.

A 55-year-old female initially underwent laparoscopic fundoplication. Due to recurrent hiatus hernia and reflux symptoms, she required revision surgery with TiMesh reinforcement 2 years later for a moderately sized HH. Fourteen years later, she presented with an upper gastrointestinal bleed secondary to gastritis, exacerbated by antiplatelet therapy. Gastroscopy revealed visible sutures and mesh at the gastric cardia with adjacent mucosal erosion (Fig. 1). Her symptoms resolved with conservative management.

### Case 3

**Figure 2:**
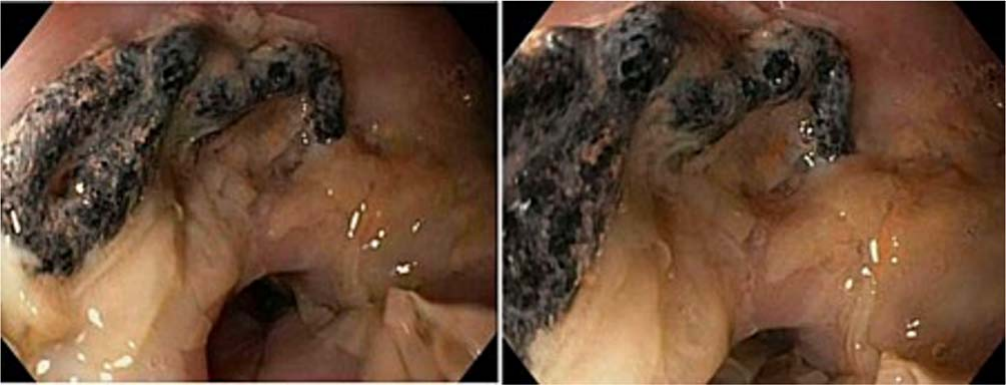
Gastroscopy images of lower third of oesophagus showing multiple linear slough partially detached in sheets from the underlying mucosa, and a 2 cm piece of eroded mesh.

A 69-year-old female with an extensive medical history, including prior colonic resection, chronic obstructive pulmonary disease (COPD) (active smoker), and Parkinson’s disease, underwent HH repair with TiMesh reinforcement. Nine years later, she underwent diagnostic gastroscopy for left upper quadrant abdominal pain. The lower 10 cm of oesophagus appeared diffusely abnormal, described as ‘sloughy oesophagus’ (Fig. 2). A 3 cm diverticulum was seen just above the cardio-oesophageal junction. At 40 cm, a 2 cm segment of surgical mesh was visible intraluminally, with an apparent point of mucosal ingress. Despite these findings, she remained asymptomatic and did not require any operative intervention.

## Discussion

These cases illustrate the low but important risk of mesh erosion following HH repair with TiMesh reinforcement. All erosions occurred years after the initial surgery and were detected incidentally during investigations for unrelated symptoms. Crucially, none required mesh explantation. Overall, the rate of mesh erosion was low (0.76%), and no early erosions were observed. The absence of routine surveillance endoscopy suggests that additional asymptomatic cases may go undetected. Nevertheless, the clinical significance of these findings appears limited, given the absence of severe complications or the need for mesh removal in this cohort. These findings may be reflected by the more favourable properties of lightweight synthetic meshes compared with heavier monofilament meshes and expanded polytetrafluoroethylene (ePTFE). It is worth noting that many case reports were published in the era prior to the use of light weight non-absorbable meshes when ePTFE and heavier polypropylene meshes were utilized.

The use of synthetic mesh in paraoesophageal hernia repair reinforcement remains contentious due to its association with risks such as erosion, stricture formation, dysphagia, and visceral injury [10, 11]. While synthetic mesh effectively reduces recurrence rates compared to suture repair [3], the potential for catastrophic erosion and obstruction of hiatal mesh is well known to foregut surgeons with the potential for oesophagogastric resection required to treat some cases [8, 12]. Higher overall complications are associated with patients who have had prior hiatal mesh repair during laparoscopic HH revision, though major visceral complications were comparable regardless of mesh use. This suggests that previous mesh placement should not preclude reoperative surgery [13]. Systematic reviews corroborate that mesh erosion, though rare, often requires surgical intervention, highlighting the need to weigh anatomical benefits against potential long-term risks [14, 15]. Meta-analyses of mesh re-enforced crural repair have been contradictory with findings for and against. A recent publication of recommendations for the treatment of large HH recommended mesh use although the lack of compelling evidence is acknowledged [16].

Long-term outcomes from other series further contextualize these findings. High patient satisfaction and durable repair efficacy have been reported with non-absorbable mesh, with endoscopic follow-up revealing no mesh-related complications in most cases [17, 18]. Laparoscopic repair of large HH using TiMesh demonstrates excellent symptomatic outcomes (patient satisfaction at 1 year and significant improvements in quality of life without worsening dysphagia scores) [19]. Biological meshes, while safer and better tolerated, are associated with higher recurrence rates, though most recurrences are asymptomatic and seldom require reoperation [20]. Alternative techniques, including falciform ligament reinforcement, have also been investigated as potential options for crural repair [21].

In conclusion, mesh erosion following non-absorbable mesh–reinforced HH repair is a recognized complication that may present many years after the index operation. In this case series, mesh erosions were identified incidentally and were not associated with severe symptoms or the need for mesh explantation. These cases highlight that mesh erosion may be clinically silent and may be managed conservatively. Awareness of this potential late complication remains important when counselling patients and interpreting endoscopic findings after HH repair.
